# Supplementary material for: Modeling glioblastoma heterogeneity as a dynamic network of cell states
Source: Mol Syst Biol. 2021 Sep 16;17(9):e10105. doi: 10.15252/msb.202010105 (PMC8444284; doi:10.15252/msb.202010105)
Supplement: Supplementary file 5 — Source Data for Figure 3 [file MSB-17-e10105-s001.zip › Figure3A_sourcedata/GSEA_3065/hallmarks_state4.GseaPreranked.1623416296068/HALLMARK_OXIDATIVE_PHOSPHORYLATION.html]

Details for gene set HALLMARK\_OXIDATIVE\_PHOSPHORYLATION[GSEA]

|  || Dataset | state4 |
| Phenotype | NoPhenotypeAvailable |
| Upregulated in class | na\_pos |
| GeneSet | HALLMARK\_OXIDATIVE\_PHOSPHORYLATION |
| Enrichment Score (ES) | 0.53495157 |
| Normalized Enrichment Score (NES) | 2.4070096 |
| Nominal p-value | 0.0 |
| FDR q-value | 0.0 |
| FWER p-Value | 0.0 |
Table: GSEA Results Summary

  

Fig 1: Enrichment plot: HALLMARK\_OXIDATIVE\_PHOSPHORYLATION      
 Profile of the Running ES Score & Positions of GeneSet Members on the Rank Ordered List

  

| PROBE | GENE SYMBOL | GENE\_TITLE | RANK IN GENE LIST | RANK METRIC SCORE | RUNNING ES | CORE ENRICHMENT || 1 | ATP6V1G1 |  |  | 47 | 0.303 | 0.0145 | Yes |
| 2 | ATP6V1F |  |  | 96 | 0.251 | 0.0250 | Yes |
| 3 | UQCR11 |  |  | 107 | 0.245 | 0.0413 | Yes |
| 4 | UQCRB |  |  | 114 | 0.241 | 0.0579 | Yes |
| 5 | ATP5F1E |  |  | 119 | 0.238 | 0.0747 | Yes |
| 6 | SLC25A6 |  |  | 147 | 0.223 | 0.0866 | Yes |
| 7 | UQCRQ |  |  | 158 | 0.218 | 0.1009 | Yes |
| 8 | COX7C |  |  | 162 | 0.216 | 0.1163 | Yes |
| 9 | GPX4 |  |  | 183 | 0.210 | 0.1283 | Yes |
| 10 | COX6B1 |  |  | 185 | 0.209 | 0.1435 | Yes |
| 11 | NDUFA3 |  |  | 229 | 0.191 | 0.1504 | Yes |
| 12 | ECH1 |  |  | 245 | 0.187 | 0.1616 | Yes |
| 13 | COX6C |  |  | 269 | 0.181 | 0.1710 | Yes |
| 14 | ATP5PO |  |  | 272 | 0.180 | 0.1839 | Yes |
| 15 | ATP5ME |  |  | 281 | 0.178 | 0.1956 | Yes |
| 16 | TIMM8B |  |  | 283 | 0.178 | 0.2085 | Yes |
| 17 | NDUFB1 |  |  | 289 | 0.175 | 0.2205 | Yes |
| 18 | ATP5F1D |  |  | 304 | 0.173 | 0.2309 | Yes |
| 19 | ETFB |  |  | 315 | 0.172 | 0.2418 | Yes |
| 20 | ATP6V0E1 |  |  | 338 | 0.168 | 0.2505 | Yes |
| 21 | DECR1 |  |  | 340 | 0.167 | 0.2625 | Yes |
| 22 | ATP5MG |  |  | 350 | 0.165 | 0.2731 | Yes |
| 23 | ATP5PD |  |  | 375 | 0.159 | 0.2808 | Yes |
| 24 | ATP5F1A |  |  | 422 | 0.148 | 0.2841 | Yes |
| 25 | NDUFB2 |  |  | 423 | 0.148 | 0.2949 | Yes |
| 26 | NDUFA2 |  |  | 441 | 0.145 | 0.3027 | Yes |
| 27 | NDUFB7 |  |  | 445 | 0.144 | 0.3128 | Yes |
| 28 | ATP5MC2 |  |  | 455 | 0.143 | 0.3218 | Yes |
| 29 | COX7A2 |  |  | 460 | 0.143 | 0.3316 | Yes |
| 30 | ATP5PF |  |  | 489 | 0.139 | 0.3372 | Yes |
| 31 | HADHB |  |  | 503 | 0.136 | 0.3450 | Yes |
| 32 | NDUFA5 |  |  | 516 | 0.134 | 0.3528 | Yes |
| 33 | ATP5MF |  |  | 519 | 0.134 | 0.3623 | Yes |
| 34 | BAX |  |  | 521 | 0.134 | 0.3719 | Yes |
| 35 | PDP1 |  |  | 554 | 0.131 | 0.3762 | Yes |
| 36 | SURF1 |  |  | 577 | 0.128 | 0.3820 | Yes |
| 37 | NDUFA8 |  |  | 604 | 0.125 | 0.3869 | Yes |
| 38 | ISCU |  |  | 608 | 0.125 | 0.3955 | Yes |
| 39 | ATP6V1E1 |  |  | 612 | 0.124 | 0.4041 | Yes |
| 40 | NDUFB5 |  |  | 614 | 0.124 | 0.4130 | Yes |
| 41 | IDH2 |  |  | 615 | 0.124 | 0.4220 | Yes |
| 42 | COX7A2L |  |  | 617 | 0.124 | 0.4309 | Yes |
| 43 | ISCA1 |  |  | 629 | 0.122 | 0.4381 | Yes |
| 44 | NDUFB4 |  |  | 649 | 0.120 | 0.4438 | Yes |
| 45 | COX5B |  |  | 677 | 0.117 | 0.4479 | Yes |
| 46 | BDH2 |  |  | 688 | 0.116 | 0.4547 | Yes |
| 47 | POLR2F |  |  | 700 | 0.114 | 0.4613 | Yes |
| 48 | NDUFS6 |  |  | 707 | 0.113 | 0.4685 | Yes |
| 49 | UQCRH |  |  | 710 | 0.113 | 0.4764 | Yes |
| 50 | VDAC2 |  |  | 795 | 0.104 | 0.4703 | Yes |
| 51 | MAOB |  |  | 799 | 0.104 | 0.4774 | Yes |
| 52 | COX4I1 |  |  | 839 | 0.101 | 0.4784 | Yes |
| 53 | CYB5R3 |  |  | 846 | 0.100 | 0.4847 | Yes |
| 54 | PHYH |  |  | 865 | 0.098 | 0.4889 | Yes |
| 55 | COX7B |  |  | 872 | 0.098 | 0.4951 | Yes |
| 56 | SDHC |  |  | 880 | 0.097 | 0.5011 | Yes |
| 57 | NDUFB3 |  |  | 883 | 0.097 | 0.5079 | Yes |
| 58 | NDUFS7 |  |  | 887 | 0.097 | 0.5145 | Yes |
| 59 | NDUFA1 |  |  | 955 | 0.092 | 0.5102 | Yes |
| 60 | NDUFA4 |  |  | 977 | 0.090 | 0.5133 | Yes |
| 61 | ACAA1 |  |  | 986 | 0.089 | 0.5185 | Yes |
| 62 | ACO2 |  |  | 1077 | 0.083 | 0.5099 | Yes |
| 63 | NDUFV1 |  |  | 1095 | 0.082 | 0.5131 | Yes |
| 64 | MDH2 |  |  | 1114 | 0.081 | 0.5161 | Yes |
| 65 | UQCR10 |  |  | 1128 | 0.080 | 0.5199 | Yes |
| 66 | TIMM13 |  |  | 1143 | 0.079 | 0.5234 | Yes |
| 67 | NDUFS4 |  |  | 1144 | 0.079 | 0.5292 | Yes |
| 68 | ATP6V1C1 |  |  | 1145 | 0.079 | 0.5350 | Yes |
| 69 | UQCRC2 |  |  | 1208 | 0.076 | 0.5303 | No |
| 70 | GLUD1 |  |  | 1295 | 0.071 | 0.5215 | No |
| 71 | ATP5PB |  |  | 1301 | 0.071 | 0.5258 | No |
| 72 | MRPL34 |  |  | 1319 | 0.070 | 0.5282 | No |
| 73 | ATP5F1B |  |  | 1379 | 0.067 | 0.5234 | No |
| 74 | ATP5F1C |  |  | 1410 | 0.066 | 0.5233 | No |
| 75 | NDUFS1 |  |  | 1453 | 0.064 | 0.5211 | No |
| 76 | NDUFC1 |  |  | 1459 | 0.063 | 0.5249 | No |
| 77 | ACAA2 |  |  | 1493 | 0.062 | 0.5241 | No |
| 78 | ATP6V1D |  |  | 1507 | 0.061 | 0.5264 | No |
| 79 | COX8A |  |  | 1583 | 0.059 | 0.5185 | No |
| 80 | ACADVL |  |  | 1600 | 0.059 | 0.5202 | No |
| 81 | IDH1 |  |  | 1691 | 0.055 | 0.5095 | No |
| 82 | HSD17B10 |  |  | 1695 | 0.055 | 0.5130 | No |
| 83 | PDHB |  |  | 1749 | 0.054 | 0.5083 | No |
| 84 | NDUFS2 |  |  | 1752 | 0.054 | 0.5119 | No |
| 85 | NDUFAB1 |  |  | 1773 | 0.053 | 0.5125 | No |
| 86 | RHOT1 |  |  | 1782 | 0.053 | 0.5151 | No |
| 87 | ALDH6A1 |  |  | 1803 | 0.052 | 0.5156 | No |
| 88 | ETFA |  |  | 1831 | 0.051 | 0.5149 | No |
| 89 | GPI |  |  | 1849 | 0.051 | 0.5159 | No |
| 90 | MPC1 |  |  | 1881 | 0.050 | 0.5144 | No |
| 91 | DLD |  |  | 1906 | 0.049 | 0.5141 | No |
| 92 | UQCRFS1 |  |  | 2090 | 0.044 | 0.4874 | No |
| 93 | OAT |  |  | 2106 | 0.044 | 0.4882 | No |
| 94 | ACADM |  |  | 2224 | 0.041 | 0.4720 | No |
| 95 | IDH3G |  |  | 2528 | 0.034 | 0.4249 | No |
| 96 | OXA1L |  |  | 2580 | 0.033 | 0.4190 | No |
| 97 | NDUFS8 |  |  | 2582 | 0.033 | 0.4213 | No |
| 98 | TIMM9 |  |  | 2592 | 0.033 | 0.4222 | No |
| 99 | ATP5MC1 |  |  | 2610 | 0.033 | 0.4218 | No |
| 100 | ECHS1 |  |  | 2628 | 0.032 | 0.4214 | No |
| 101 | NDUFV2 |  |  | 2750 | 0.030 | 0.4038 | No |
| 102 | COX15 |  |  | 2771 | 0.029 | 0.4027 | No |
| 103 | SLC25A12 |  |  | 2889 | 0.027 | 0.3855 | No |
| 104 | LDHB |  |  | 2930 | 0.026 | 0.3808 | No |
| 105 | COX11 |  |  | 2983 | 0.025 | 0.3742 | No |
| 106 | MGST3 |  |  | 2993 | 0.025 | 0.3745 | No |
| 107 | PHB2 |  |  | 3200 | 0.021 | 0.3424 | No |
| 108 | SLC25A3 |  |  | 3260 | 0.020 | 0.3341 | No |
| 109 | HADHA |  |  | 3376 | 0.016 | 0.3165 | No |
| 110 | FXN |  |  | 3381 | 0.016 | 0.3170 | No |
| 111 | PRDX3 |  |  | 3387 | 0.016 | 0.3173 | No |
| 112 | SUCLG1 |  |  | 3497 | 0.012 | 0.3004 | No |
| 113 | TOMM22 |  |  | 3575 | 0.010 | 0.2885 | No |
| 114 | SLC25A4 |  |  | 3579 | 0.010 | 0.2887 | No |
| 115 | RETSAT |  |  | 3588 | 0.009 | 0.2881 | No |
| 116 | MDH1 |  |  | 3601 | 0.009 | 0.2868 | No |
| 117 | MRPS15 |  |  | 3653 | 0.007 | 0.2790 | No |
| 118 | NDUFS3 |  |  | 3667 | 0.006 | 0.2773 | No |
| 119 | ACAT1 |  |  | 3723 | 0.004 | 0.2686 | No |
| 120 | COX17 |  |  | 3749 | 0.003 | 0.2648 | No |
| 121 | COX6A1 |  |  | 3753 | 0.003 | 0.2645 | No |
| 122 | SLC25A11 |  |  | 3808 | 0.000 | 0.2557 | No |
| 123 | ECI1 |  |  | 3888 | -0.003 | 0.2430 | No |
| 124 | ATP6V1H |  |  | 3936 | -0.006 | 0.2357 | No |
| 125 | CYB5A |  |  | 3954 | -0.007 | 0.2334 | No |
| 126 | NQO2 |  |  | 3986 | -0.009 | 0.2290 | No |
| 127 | FDX1 |  |  | 4016 | -0.010 | 0.2250 | No |
| 128 | COX5A |  |  | 4019 | -0.010 | 0.2254 | No |
| 129 | NDUFA6 |  |  | 4074 | -0.014 | 0.2176 | No |
| 130 | NDUFB8 |  |  | 4100 | -0.015 | 0.2146 | No |
| 131 | IDH3A |  |  | 4114 | -0.016 | 0.2137 | No |
| 132 | UQCRC1 |  |  | 4194 | -0.022 | 0.2024 | No |
| 133 | NDUFB6 |  |  | 4197 | -0.022 | 0.2036 | No |
| 134 | MRPL11 |  |  | 4290 | -0.028 | 0.1906 | No |
| 135 | HSPA9 |  |  | 4311 | -0.030 | 0.1896 | No |
| 136 | ATP6AP1 |  |  | 4313 | -0.030 | 0.1916 | No |
| 137 | ATP5MC3 |  |  | 4346 | -0.032 | 0.1887 | No |
| 138 | MRPS11 |  |  | 4528 | -0.046 | 0.1625 | No |
| 139 | CYC1 |  |  | 4648 | -0.055 | 0.1470 | No |
| 140 | VDAC1 |  |  | 4748 | -0.064 | 0.1355 | No |
| 141 | SDHA |  |  | 4873 | -0.073 | 0.1205 | No |
| 142 | CYCS |  |  | 4894 | -0.075 | 0.1227 | No |
| 143 | VDAC3 |  |  | 4979 | -0.081 | 0.1149 | No |
| 144 | IMMT |  |  | 5023 | -0.084 | 0.1140 | No |
| 145 | NDUFA9 |  |  | 5088 | -0.091 | 0.1102 | No |
| 146 | MRPL15 |  |  | 5251 | -0.106 | 0.0915 | No |
| 147 | RHOT2 |  |  | 5253 | -0.106 | 0.0991 | No |
| 148 | POR |  |  | 5407 | -0.119 | 0.0828 | No |
| 149 | NDUFC2 |  |  | 5475 | -0.128 | 0.0812 | No |
| 150 | SLC25A5 |  |  | 5628 | -0.148 | 0.0672 | No |
| 151 | ATP6V0B |  |  | 5961 | -0.226 | 0.0294 | No |
| 152 | LDHA |  |  | 6057 | -0.272 | 0.0337 | No |
Table: GSEA details [plain text format]

  

Fig 2: HALLMARK\_OXIDATIVE\_PHOSPHORYLATION: Random ES distribution      
 Gene set null distribution of ES for **HALLMARK\_OXIDATIVE\_PHOSPHORYLATION**
